# Rare Appendicular Pathologies: Diagnostic Challenges, Surgical Management, and Outcomes in a Retrospective Tertiary-Center Cohort with Literature Review

**DOI:** 10.3390/jcm15093226

**Published:** 2026-04-23

**Authors:** Raluca-Cristina Ailioaie, Vlad Fagarasan, Catalin Ciuce, Razvan Scurtu, George Dindelegan

**Affiliations:** 1 Department of Surgery, Iuliu Hatieganu University of Medicine and Pharmacy, 400347 Cluj-Napoca, Romania; raluca.apostu@umfcluj.ro (R.-C.A.); fagarasan.vlad@elearn.umfcluj.ro (V.F.); george.dindelegan@umfcluj.ro (G.D.); 2 First Surgical Clinic, Emergency County University Hospital, 400347 Cluj-Napoca, Romania; catalinciuce@gmail.com

**Keywords:** appendix, rare appendicular pathology, low-grade appendiceal mucinous neoplasm, adenocarcinoma, diverticulum, stump appendicitis

## Abstract

**Background:** Rare appendicular pathologies (RAP) are uncommon clinical entities with important diagnostic and therapeutic implications. These conditions frequently mimic acute appendicitis, yet they may require different operative strategies and, in selected cases, oncological management. **Methods:** We performed a retrospective cohort study including all patients who underwent surgery with the intention of performing an appendectomy at the First Surgical Clinic, Emergency County University Hospital of Cluj-Napoca, between 2018 and 2021. During this interval, 330 appendectomies were performed. Patients with a histopathological diagnosis of RAP were included. Clinical, imaging, surgical, histopathological, postoperative, and follow-up data were analyzed, with particular attention to the preoperative diagnostic work-up and imaging-based suspicion of rare appendicular pathology. **Results:** Ten patients (3.03%) were diagnosed with RAP, including low-grade appendiceal mucinous neoplasm (LAMN; n = 5), mucinous cystadenoma (n = 2), appendiceal adenocarcinoma (n = 1), appendicular diverticulum (n = 1), and stump appendicitis (n = 1). Computed tomography was the main diagnostic modality, particularly in patients with atypical presentation or suspicion of complicated or neoplastic appendiceal disease, while magnetic resonance imaging and colonoscopy provided additional information in selected cases. Preoperative suspicion of a rare or neoplastic appendiceal pathology was achieved in 70% of patients. Laparoscopic appendectomy was performed in 6 patients, open appendectomy in 1 patient, open ileocecal resection in 1 patient, open right hemicolectomy in 1 patient, and laparoscopic right hemicolectomy in 1 patient. Histopathological examination confirmed the diagnosis in all cases. Immediate postoperative outcomes were favorable, without perioperative mortality or major complications; during follow-up, the patient with adenocarcinoma required oncological treatment and resection of a local recurrence 1 year after surgery. **Conclusions:** RAP represent a small but clinically significant subset of appendiceal disease. Structured preoperative imaging, intraoperative recognition of atypical findings, and an individualized surgical strategy are essential for optimal outcomes and appropriate oncological management.

## 1. Introduction

Appendicular pathology is dominated by acute appendicitis; however, rare appendicular pathologies (RAP) represent an uncommon but clinically relevant subgroup that may mimic typical appendicitis or other abdominal conditions and may therefore be misdiagnosed or managed inappropriately. RAP encompass benign entities with atypical presentation, such as appendiceal diverticula and stump appendicitis, as well as neoplastic disease ranging from mucinous lesions to adenocarcinoma, for which the correct intraoperative strategy and postoperative oncological planning are essential [1,2,3].

Neoplastic appendiceal mucinous lesions are particularly important because inaccurate diagnosis or iatrogenic rupture may result in peritoneal dissemination and pseudomyxoma peritonei, with major implications for long-term morbidity and survival. Contemporary nomenclature and classification have evolved, and diagnostic and therapeutic decisions increasingly rely on standardized pathological frameworks and guideline recommendations [4,5,6,7].

Given their atypical clinical features, imaging is central to raising diagnostic suspicion and planning surgery. Cross-sectional imaging descriptions of appendiceal mucocele, as well as computed tomography (CT)-based tools developed to predict an underlying tumor in inflammatory settings, support a structured approach in doubtful presentations. Patient age and appendiceal diameter on CT have also been associated with malignancy risk in appendicitis cohorts [8,9,10].

Discrepancies between clinical and pathological diagnosis have been reported, and CT-pathology correlations have described imaging patterns of primary appendiceal neoplasms presenting as appendicitis, including features that help differentiate neoplastic mucinous lesions from inflammatory disease. These observations reinforce the need to integrate imaging findings, operative assessment, and histopathology in a cohesive diagnostic pathway [11,12,13,14,15].

In this context, we conducted a retrospective cohort study of appendectomies performed at a tertiary surgical center to quantify the institutional incidence of RAP, describe the diagnostic work-up, and analyze surgical management and outcomes. The overarching goal was to improve recognition and decision-making when these pathologies are suspected preoperatively or encountered unexpectedly as intraoperative surprises.

## 2. Materials and Methods

This retrospective cohort study was conducted at the First Surgical Clinic, Emergency County University Hospital of Cluj-Napoca. All patients who underwent surgery with the intention of appendectomy between 2018 and 2021 were identified. During this period, 330 appendectomies were performed.

Patients diagnosed with RAP on histopathological examination were included. RAP were defined as appendiceal mucinous neoplasms, mucinous cystadenomas, adenocarcinoma, appendicular diverticula, and stump appendicitis.

Collected variables included demographic characteristics, clinical presentation, preoperative imaging findings, intraoperative findings, type of surgical intervention, histopathological diagnosis, postoperative outcomes, and follow-up.

Preoperative evaluation followed the institutional diagnostic pathway for patients presenting with suspected acute appendiceal pathology. Clinical examination and laboratory tests were performed in all patients, and abdominal ultrasonography was generally used as the first-line imaging modality. Computed tomography (CT) was indicated in patients with atypical or prolonged symptoms, inconclusive ultrasonography, suspicion of complicated appendicitis, suspicion of a mass-forming or neoplastic appendiceal lesion, or when a broader differential diagnosis had to be considered. Magnetic resonance imaging (MRI) and colonoscopy were used selectively in cases requiring further characterization of suspected mucinous, cecal-based, or otherwise atypical lesions.

Because this was a retrospective study focused on histopathologically confirmed rare appendicular pathologies, serum tumor markers were not systematically available for all patients and were therefore not included as a standard component of the preoperative work-up.

Surgical management consisted of laparoscopic or open appendectomy in localized disease, ileocecal resection in selected cases, and open or laparoscopic right hemicolectomy in patients with suspected or confirmed malignant involvement or appendiceal base/cecal extension.

The definitive diagnosis was established through histopathological examination of the surgical specimens.

## 3. Results

Out of 330 appendectomies performed during the study period, 10 patients (3.03%) were diagnosed with RAP.

The distribution was as follows: LAMN, 5 cases (50%); mucinous cystadenoma, 2 cases (20%); appendiceal adenocarcinoma, 1 case (10%); appendicular diverticulum, 1 case (10%); and stump appendicitis, 1 case (10%).

The mean age was 50 years (range, 26–84 years), with a predominance of female patients. Clinical presentation varied from acute right lower quadrant pain to chronic abdominal symptoms and incidental intraoperative findings. Some patients presented with nonspecific symptoms, whereas others were investigated for bowel obstruction, suspected intussusception, or a possible intra-abdominal mass (Table 1).

**Figure 1:**
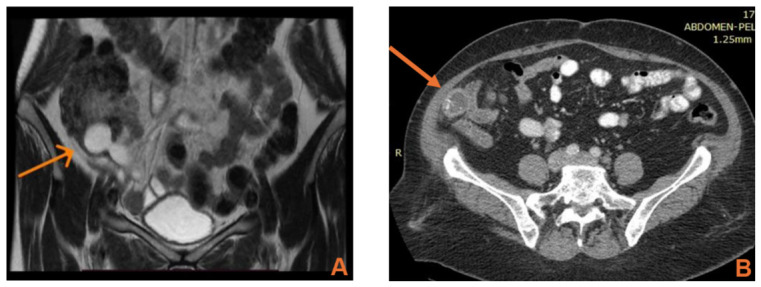

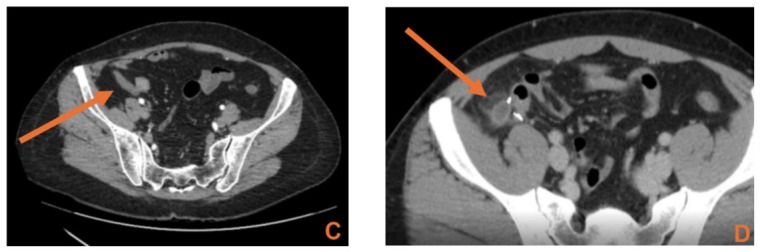
MRI and CT findings in rare appendicular pathologies. (**A**) Pelvic MRI showing a dilated appendiceal base suggestive of a mucocele (arrow). (**B**) CT scan of a mucinous cystadenoma demonstrating appendiceal dilatation with mural calcifications (arrow; R marks the right side of the patient). (**C**) CT scan showing nonspecific findings in appendicular diverticulum (arrow). (**D**) CT scan demonstrating stump appendicitis, with previously placed clips at the appendiceal base and appendicular artery (arrow).

CT was performed in seven patients and represented the primary diagnostic modality. CT was mainly used in patients with atypical clinical presentation, prolonged symptom duration, inconclusive first-line ultrasonography, suspected complication, or imaging suspicion of a mucinous or neoplastic appendiceal lesion. MRI and colonoscopy provided additional diagnostic information in selected cases, particularly when a mucinous or otherwise neoplastic lesion was suspected (Table 1, Figure 1).

**Figure 2:**
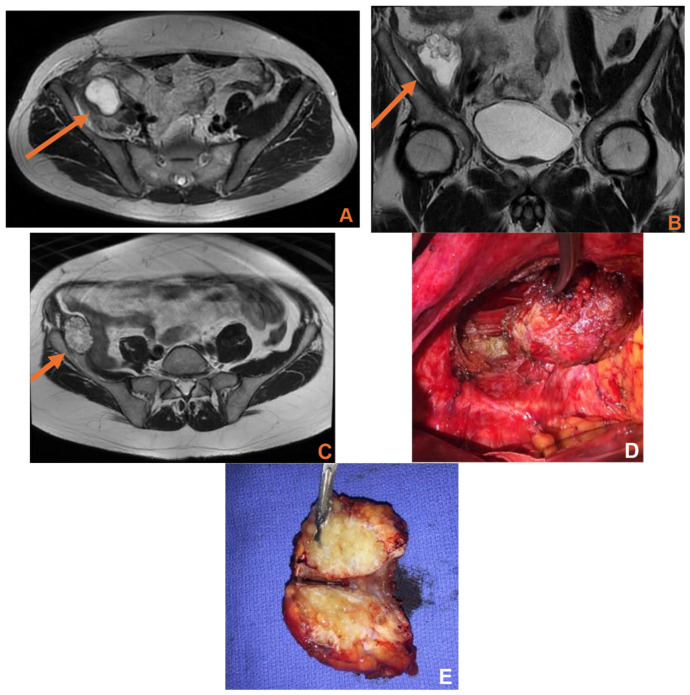
Appendiceal adenocarcinoma. (**A**–**C**) MRI demonstrating a fluid collection in the right iliac fossa associated with a tumoral mass and invasion of the iliopsoas muscle (arrow). (**D**,**E**) Intraoperative view of the local recurrence, which was completely resected.

Preoperative diagnosis or strong suspicion of a rare appendicular pathology was achieved in 70% of patients. This preoperative suspicion was based primarily on imaging findings and atypical clinical presentation rather than on systematic biomarker assessment (Figure 2).

**Figure 3:**
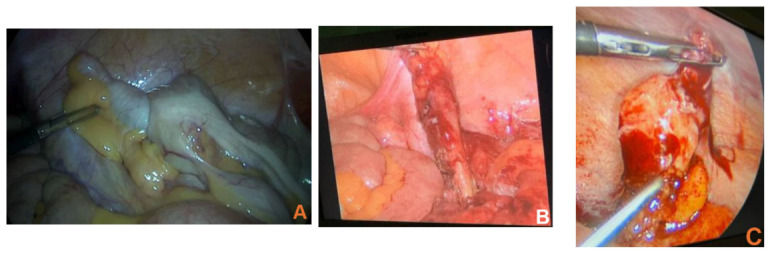
Intraoperative findings in rare appendicular pathologies. (**A**) Appendiceal mucocele with cecal intussusception. (**B**) Appendicular diverticulum. (**C**) A 2 cm stump appendicitis with the residual mesoappendix.

Laparoscopic appendectomy was performed in 6 patients and open appendectomy in 1 patient with localized pathology. Open ileocecal resection was required in 1 patient, open right hemicolectomy in 1 patient because of confirmed malignant disease, and laparoscopic right hemicolectomy in 1 patient when involvement of the appendiceal base and cecum was identified (Table 2, Figure 3).

Histopathological examination confirmed LAMN, mucinous cystadenoma, adenocarcinoma of the appendix, appendicular diverticulum, and stump appendicitis (Table 2). No perioperative mortality or major postoperative complications were recorded. Follow-up extended to 5 years. Patients with neoplastic pathology were referred for oncological follow-up according to institutional protocols. The patient with adenocarcinoma was lost to follow-up after 1 year from the last surgery.

A structured summary of the main imaging features that may help differentiate typical acute appendicitis from rare appendicular pathologies is provided in Table 3.

## 4. Discussion

### 4.1. Overall Incidence and Diagnostic Implications

In our cohort of 330 appendectomy-intended operations, RAP were identified in 10 patients (3.03%). This proportion is consistent with the low incidence of appendiceal neoplasms and other rare appendicular entities reported in appendectomy series, while also highlighting their clinical relevance because management may differ substantially from standard treatment for uncomplicated appendicitis [1,2,3].

Our data also confirms the diagnostic heterogeneity of RAP. As summarized in Table 1, symptomatology ranged from chronic or intermittent right iliac fossa pain in several mucinous lesions, to incidental intraoperative detection of LAMN during surgery for bowel obstruction, to a long-standing inguinal/iliopsoas infectious-mass presentation caused by appendiceal adenocarcinoma, and to a typical post-appendectomy inflammatory syndrome in the stump appendicitis case. This broad clinical spectrum mirrors the literature showing that RAP may present as apparent acute appendicitis, as a chronic indolent process, or as a mass-forming condition discovered only after extended imaging or during surgery [11,12,13,14,15].

Preoperative suspicion was achieved in 70% of our patients, but the tables also show why diagnosis remains difficult: several cases required multiple imaging investigations before the pathology was sufficiently characterized. CT was the main diagnostic tool in our cohort and was particularly informative in patients with atypical presentation, prolonged symptom duration, inconclusive initial ultrasonography, or suspicion of a mucinous or neoplastic lesion, whereas MRI refined lesion characterization in selected patients and colonoscopy provided complementary information in cases with appendiceal-base or cecal involvement. This pattern is in line with published studies showing that cross-sectional imaging has a central role in distinguishing inflammatory from neoplastic appendiceal disease and in identifying features associated with malignancy risk [8,9,10,11,12,13,14,15]. Serum tumor markers were not systematically available in this retrospective cohort; therefore, preoperative suspicion relied mainly on imaging findings and clinical presentation rather than on biomarker profiling. For practical purposes, the distinguishing imaging features between typical acute appendicitis and the main RAP entities encountered in our cohort are summarized in Table 3.

Published series report appendiceal neoplasms in up to 2.14% of appendectomy specimens, whereas appendiceal mucoceles account for approximately 0.1–1.4% of appendiceal specimens. Within mucoceles, simple mucocele has been reported in about 29% of cases, mucosal hyperplasia and appendiceal mucinous neoplasms in 31–34%, and mucinous adenocarcinoma in roughly 5%. These figures help contextualize our 3.03% RAP rate, which also includes non-neoplastic entities such as diverticulum and stump appendicitis and therefore reflects a broader clinically relevant spectrum than appendiceal tumors alone [2,3,4].

The literature also emphasizes that preoperative diagnosis remains imperfect, with reported success rates of only 15–29% in chronic presentations and approximately 7.5% in acute settings. In selected patients, serum tumor markers such as carcinoembryonic antigen (CEA), CA19-9, and CA125 may support suspicion of an underlying mucinous neoplasm or adenocarcinoma, and PSOGI recommendations include at least CEA and CA19-9 in the work-up of appendiceal mucinous tumors because elevated values correlate with recurrence risk and prognosis. These biomarkers were not systematically available in our retrospective cohort, but their potential value is particularly relevant for cases such as 3, 5, 6, 7, and 8, in which imaging raised concern for neoplastic disease [3,4,6].

Beyond its general diagnostic value, CT can provide discriminative clues that are also reflected in our tables, including appendiceal diameter greater than 10–15 mm, mural calcifications, focal or asymmetric wall abnormality, a mass-like aspect, and the absence of an appendicolith. Monsonis et al. proposed a CT-based score in inflammatory settings, while Naar et al. showed a higher malignancy risk in patients older than 40 years with an appendiceal diameter greater than 10 mm. Marotta et al. further observed that distal dilatation with a preserved proximal segment and no appendicolith strongly suggests an underlying neoplasm. In our cohort, several mucinous cases showed exactly these kinds of atypical features rather than a classic appendicitis pattern, which explains why cross-sectional imaging changed the operative strategy [9,10,14].

### 4.2. Mucinous Cystadenoma and Low-Grade Appendiceal Mucinous Neoplasm

The largest subgroup in our series was represented by mucinous lesions, namely 2 mucinous cystadenomas (cases 1 and 2) and 5 LAMN cases (cases 3–7) (Table 1 and Table 2). The literature emphasizes that mucinous appendiceal lesions frequently mimic inflammatory disease but require careful handling because rupture may lead to peritoneal dissemination and pseudomyxoma peritonei. The WHO classification, PSOGI/EURACAN recommendations, and related guideline frameworks have refined terminology and management principles for these lesions [4,5,6,7].

They distinguish serrated and hyperplastic lesions from LAMN, HAMN, and mucinous adenocarcinoma, and they emphasize that even low-grade lesions may become clinically important if mucin or neoplastic epithelium disseminates into the peritoneal cavity. Pseudomyxoma peritonei is likewise stratified into acellular mucin, low-grade mucinous carcinoma peritonei, high-grade mucinous carcinoma peritonei, and high-grade mucinous carcinoma peritonei with signet-ring cells, with major prognostic implications. None of our mucinous cases developed pseudomyxoma peritonei during follow-up, which is reassuring and consistent with the intact resection strategy documented in Table 2 [5,6,7].

In our patients, the mucinous-spectrum cases showed substantial clinical variability. Cases 1, 2, 3, and 7 presented with localized but non-specific or chronic pain, case 5 was investigated during a gynecologic work-up, case 6 raised suspicion of ileocecal intussusception, and case 4 was discovered incidentally during surgery for bowel obstruction due to an incarcerated obturator hernia (Table 1). These observations support the literature that appendiceal mucinous lesions often deviate from the classical clinical picture of acute appendicitis and are therefore vulnerable to delayed or incorrect diagnosis [8,11,12,13,14,15,16].

Imaging findings in our series were concordant with the literature. MRI characterized fluid-filled, well-delimited lesions in cases 1 and 5, while CT demonstrated appendiceal dilatation, mucinous content, appendiceal base involvement, or a target-like appearance in cases 2, 3, 5, 6, and 7 (Table 1). These case-level findings resemble the published imaging descriptions of appendiceal mucocele and the CT features that may suggest an underlying mucinous neoplasm rather than pure inflammation [8,9,10,11,12,13,14,15,16].

The imaging details described in the literature align closely with the findings summarized in Table 1. On CT, appendiceal mucoceles are typically described as well-encapsulated, low-attenuation round or tubular cystic masses adjacent to the cecum; proposed discriminatory criteria include a luminal diameter above 13–15 mm, mural thickness greater than 6 mm, and mural calcifications, although calcifications are not present in all cases. Ultrasonography may reveal the characteristic onion-skin sign, whereas colonoscopy may show a volcano sign or mound-like elevation at the appendiceal orifice and is also useful for detecting synchronous colorectal lesions. MRI usually demonstrates variable T1 signal and T2 hyperintensity and is especially helpful when rupture or extraluminal mucin is suspected. In our series, MRI and colonoscopy were particularly valuable in selected cases with appendiceal-base or cecal involvement, while CT remained the key modality for operative planning [1,3,4,8,11,12,15].

Emerging studies suggest that CT-based radiomics models and 18F-FAPI PET/CT may further improve the differentiation and staging of appendiceal mucinous neoplasms [17,18,19,20]. Such advanced techniques were not used in our cohort; however, the information summarized in Table 1 shows that conventional CT and MRI already provided enough preoperative anatomical detail in most of our mucinous cases to justify an oncologically cautious operative strategy.

Histopathological examination remained decisive. In the mucinous cystadenoma and LAMN cases, Table 2 documents the hallmarks of low-grade mucinous pathology, including a markedly dilated mucin-filled lumen, thinned or fibrotic wall, low-grade epithelial atypia, mural or submucosal dissecting acellular mucin with a pushing rather than infiltrative pattern, and absence of overt invasive carcinoma in cases 1–7. These findings are consistent with contemporary pathological frameworks, while also illustrating the interpretative challenges discussed in the literature, particularly the need to distinguish true LAMN from post-inflammatory mucosal hyperplasia or diverticular-associated mucinous changes [5,21,22].

Features favoring a non-neoplastic post-inflammatory process include preserved wall layers away from perforation sites, intact muscularis mucosae, mature goblet cells in the surface epithelium, and a clinical-radiologic context suggestive of acute inflammation. Our diagnoses in cases 1–7 were supported by the combination of morphology and the non-classic inflammatory presentation summarized in the tables [1,4,21,22].

Our operative management also paralleled the literature. Most localized lesions were treated by minimally invasive appendectomy (cases 1, 2, and 7), whereas limited extension of resection was used when the base or cecal wall was involved (case 3), and more extensive surgery was chosen in the presence of broad appendiceal base involvement or invagination/intussusception-like appearance (cases 5 and 6) (Table 2). This individualized approach is concordant with reports indicating that appendectomy with negative margins may be curative for intact localized disease, while right hemicolectomy or ileocecal resection should be reserved for selected patients in whom the appendiceal base, cecum, or oncologic risk profile justifies a wider resection [23,24,25,26,27,28].

Several authors have reported no clear survival advantage for routine right hemicolectomy in low-grade localized mucinous tumors, which supports the selective rather than systematic extension of resection used in our series. Careful assessment of the appendiceal base, atraumatic handling, and specimen retrieval in an endobag remain essential to avoid iatrogenic rupture [23,24,25,26].

This caution is justified because tumor rupture, whether spontaneous or iatrogenic, may result in peritoneal dissemination and pseudomyxoma peritonei. Reported risks of rupture and peritoneal spread range between 5% and 15%, and once peritoneal mucinous disease is present, staging and treatment may require multidisciplinary evaluation, imaging reassessment, and, in selected patients, cytoreductive surgery with HIPEC. Our favorable follow-up without pseudomyxoma peritonei supports the adequacy of the operative strategy used for the intact mucinous lesions in Table 2 [1,3,24,26].

The laparoscopic approach was feasible and safe in most of our mucinous cases, provided that careful manipulation and specimen extraction principles were respected. This again mirrors comparative and multicenter studies supporting laparoscopy in selected appendiceal mucocele and LAMN cases, as long as rupture is avoided [27,28].

### 4.3. Appendiceal Adenocarcinoma

Our single adenocarcinoma case (case 8) illustrates the aggressive end of the appendiceal pathology spectrum. Preoperative imaging demonstrated a right iliopsoas abscess extending into the inguinal canal, persistent collections, and findings highly suggestive of a mucinous appendiceal malignancy. Intraoperatively, the ceco-appendiceal tumor invaded the iliopsoas muscle and was adherent to the testicular vessels, with nodal disease and purulent tumor-associated debris; treatment required open right hemicolectomy, iliopsoas resection, and hilar node biopsy (Table 2). Final pathology showed mucinous adenocarcinoma with >50% extracellular mucin, vascular emboli, and an R1 margin, and the patient subsequently required XELOX chemotherapy and resection of a 1-year local recurrence (Table 2).

These findings are in line with the literature showing that appendiceal adenocarcinoma is rare, frequently diagnosed at an advanced stage, and biologically distinct from colorectal cancer. Published case reports and reviews have also described unusual metastatic or locoregional patterns that may initially mimic abscesses or other inflammatory processes [29,30,31]. Our case strongly supports this observation, as the presenting syndrome was not typical appendicitis but a prolonged inguinal and iliopsoas septic-mass pattern.

The broader literature further shows that appendiceal adenocarcinoma differs biologically from colorectal adenocarcinoma. Integrated clinico-molecular analyses have demonstrated distinct mutation profiles and different relationships between grade, molecular alterations, and prognosis, with tumor grade often outperforming individual somatic mutations in predicting outcome. Histologically, mucinous, non-mucinous, and signet-ring cell subtypes are recognized, with the poorest survival reported for signet-ring cell tumors. These data help explain why our patient’s adverse pathological features—locally advanced disease, vascular emboli, positive margin, and early recurrence—portended an aggressive course despite multimodal treatment [29,31].

The extent of surgery in this patient was guided by local invasion and oncologic suspicion, which is consistent with contemporary guidelines and large-database analyses emphasizing individualized surgical planning according to histologic subtype, tumor spread, and resection margins [32,33]. In high-risk situations, perioperative strategies such as cytoreductive approaches and HIPEC have been discussed, although these interventions must be balanced against substantial morbidity [34,35]. Although our patient did not undergo HIPEC, the adverse pathological features and early recurrence recorded in Table 2 underscore the poor oncologic potential of this entity.

From a practical standpoint, preoperative colonoscopic evaluation should be considered in adenocarcinoma because synchronous colorectal lesions have been reported in up to 42% of cases. Treatment is stage-adapted and may range from appendectomy with en bloc mesoappendix resection in selected localized low-grade lesions to right hemicolectomy with lymphadenectomy, systemic chemotherapy, and, for peritoneal disease, cytoreductive surgery and HIPEC. For perforated or high-risk tumors, some authors advocate systematic second-look strategies because recurrence is frequent and peritoneal metastases may be occult at the initial operation. Our case exemplifies this oncologic behavior, as local recurrence developed within 1 year despite resection and XELOX chemotherapy. However, long-term interpretation of this patient’s oncologic course remains limited because follow-up information beyond 1 year after the last operation was not available [15,30,33,34,35].

Rare reports of adenocarcinoma arising in an appendiceal stump further demonstrate that previous appendectomy does not completely eliminate the possibility of appendiceal-origin malignancy [36]. Even though our post-appendectomy case was inflammatory rather than malignant, this literature is relevant because it expands the differential diagnosis in patients presenting with stump-related pathology.

### 4.4. Appendicular Diverticulum

The patient with appendicular diverticulum (case 10) presented with right flank pain radiating to the right iliac fossa and abdominal bloating for 2 weeks. As shown in Table 1, the preoperative work-up was complex and sequential, including ultrasonography, hydrosonography, pelvic CT, colonoscopy, and repeat CT, each suggesting different possibilities ranging from cecal inflammation to appendiceal wall thickening and an adjacent nodule. Laparoscopic appendectomy finally established the diagnosis, and histopathology revealed ulcerated mesoappendiceal diverticula with suppuration extending into the adjacent fat (Table 2).

This case closely reflects the literature, which describes appendicular diverticulitis as uncommon, frequently atypical, and often difficult to recognize before surgery [37]. The meta-analytic association between appendiceal diverticulosis and neoplasia is also clinically important [38]. In this context, our case supports a low threshold for appendectomy and meticulous pathological examination whenever diverticular appendiceal disease is suspected or identified.

Appendicular diverticulosis is itself rare, accounting for approximately 0.004–2% of appendectomy specimens, but it is clinically important because of its association with perforation and neoplasia. Acquired pseudodiverticula are more common than true congenital diverticula and are usually located on the mesenteric border of the distal appendix; they carry a perforation rate reported to be as high as 30–66%, clearly exceeding that of routine appendicitis. A practical pathological classification distinguishes diverticulitis with a normal appendix, diverticulitis with appendicitis, non-inflamed diverticulum with appendicitis, and non-inflamed diverticulum with a normal appendix. Our case, in which imaging was equivocal and the final diagnosis relied on pathology, is fully consistent with this literature and reinforces the value of appendectomy plus meticulous histological assessment [37,38].

### 4.5. Stump Appendicitis

Our stump appendicitis case (case 9) presented with epigastric pain migrating to the right iliac fossa, anorexia, and localized peritonism. CT showed a 22 × 12 mm tubular stump-like structure with mesenteric fat stranding and free fluid, and laparoscopic re-intervention confirmed an approximately 2 cm fibrin-coated phlegmonous appendiceal stump (Table 1 and Table 2). Histopathology documented marked mucosal ulceration, intramural abscesses, and dense acute inflammation.

These findings correspond closely to published systematic analyses and case series, which show that stump appendicitis is a rare but clinically important post-appendectomy complication, often diagnosed late and associated with a high perforation risk when clinical suspicion is low. CT is consistently reported as the most useful diagnostic modality, and completion appendectomy remains the standard treatment, frequently feasible by laparoscopy [39,40,41]. Our case reproduces this pattern almost exactly and supports the need to consider stump appendicitis in any patient with right lower quadrant pain and a previous appendectomy history.

The literature also provides useful operative lessons for stump appendicitis. The interval from the initial appendectomy to stump appendicitis may range from a few days to as long as 50 years, and the most consistently implicated technical risk factor is an excessively long appendiceal stump, usually greater than 5 mm; some authors recommend leaving less than 3 mm when feasible. Although stump appendicitis was historically considered more common after laparoscopy, more recent series suggest that the laparoscopic approach itself is not an independent risk factor. Completion appendectomy remains the standard treatment and can usually be performed laparoscopically, whereas ileocecal resection may be necessary in complicated or perforated cases. Rare nonoperative or endoscopic alternatives have been reported, but they remain exceptional [39,40,41].

### 4.6. Study Limitations

The main limitations of this study are the small sample size and the retrospective, single-center design. Only 10 RAP cases were identified, which reflects the rarity of these entities but also limits the statistical strength of the analysis and restricts the generalizability of the findings. In addition, because the study included only patients who underwent surgery with the intention of appendectomy and had histopathologically confirmed RAP, a degree of selection bias cannot be excluded. Another limitation is that one patient with appendiceal adenocarcinoma was lost to follow-up after 1 year following the last operation, which may limit the interpretation of long-term oncologic outcomes and may underestimate late recurrence or progression. Nevertheless, the study provides clinically relevant insights because the case-level analysis highlights the heterogeneity of presentation, imaging findings, and surgical management across several distinct rare appendicular entities.

### 4.7. Clinical Implications

Early recognition of RAP is essential to ensure the correct extent of surgery and appropriate oncological management. In practical terms, the comparison between the literature and our tables suggests that CT should be obtained in all doubtful appendiceal presentations, MRI and colonoscopy may clarify selected mucinous or cecal-base lesions, and definitive management must remain individualized according to intraoperative findings and histopathology.

## 5. Conclusions

Rare appendicular pathologies represented 3.03% of appendectomy-intended operations in our series and comprised a heterogeneous group of entities with important diagnostic, surgical, and oncologic implications. Although uncommon, these lesions should be considered whenever the clinical picture is atypical or imaging findings are not fully consistent with typical acute appendicitis.

In such situations, cross-sectional imaging, particularly CT, plays a key role in raising preoperative suspicion and in guiding the extent of surgery. MRI and colonoscopy may provide additional value in selected patients with suspected mucinous, appendiceal-base, or cecal lesions.

Ultimately, optimal management of RAP depends on careful preoperative assessment, intraoperative recognition of atypical appendiceal findings, and an individualized surgical strategy based on the suspected pathology and oncologic risk. Greater awareness of these rare entities may help avoid inadequate treatment, prevent iatrogenic dissemination, and improve patient outcomes.

## Figures and Tables

**Table 1:** Baseline characteristics, presentation, and preoperative work-up.

Case	Age, y	Sex	Final Diagnosis	Presentation	Pre-Op Work-Up
1	26	F	Mucinous cystadenoma	RIF pain radiating posteriorly.	US: 5.23 × 1.56 cm elongated avascular transonic lesion with heterogeneous component. MRI: well-defined right paramedian intrapelvic fluid-filled lesion, 58.6 × 17.6 mm.
2	64	F	Mucinous cystadenoma	RLQ pain for 4 months, weight loss, intermittent diarrhea.	CT: suspected appendiceal mucocele.
3	44	M	LAMN, pTis	Chronic moderate RIF pain (~1 year).	US: bilocular right iliac lesion. Colonoscopy: ulcerative colitis. CT: 19 mm appendiceal dilatation over 35 mm at the cecal base, likely mucin; distal retrocecal appendix 6 mm; minimal periappendiceal fluid and 14 × 7 mm collection.
4	84	F	LAMN	Bowel obstruction: colicky pain, vomiting, absent gas/stool passage.	US: dilated fluid-filled bowel loops with to-and-fro peristalsis, suggestive of obstruction.
5	49	F	LAMN, pTis	Gynecologic work-up for RIF pain.	MRI: dilated appendix (27 mm at base; length 38 mm) with mucinous/fluid content, thin wall, no periappendiceal inflammation; no pelvic nodes. Colonoscopy: 13 mm lesion at appendiceal orifice with normal mucosa.
6	40	F	LAMN	6–8 weeks intermittent colicky RIF pain, nausea, vomiting; partial response to conservative therapy.	US: suspected myxoglobulosis/fecalith. CT: target sign in RIF, possible ileocecal intussusception; appendiceal mucocele/cyst vs. ileal duplication cyst.
7	56	M	LAMN	Low-intensity but persistent RIF pain.	CT: appendiceal base dilatation, minimal periappendiceal fluid, mesenteric lymphadenopathy.
8	45	M	Adenocarcinoma, pT4bN0L0V1R1	Fever, right inguinal pain, limp for 7 months.	CT: right iliopsoas abscess extending into the inguinal canal; drainage/biopsy showed mucinous adenocarcinoma of colonic origin. Repeat CT: appendiceal tumor, mesenteric and hepatic hilum nodes, persistent abscess. MRI: highly suggestive of mucinous appendiceal adenocarcinoma.
9	31	M	Stump appendicitis	Epigastric pain migrating to RIF, anorexia, localized peritonism.	CT: 22 × 12 mm tubular stump-like structure with mesenteric fat stranding and free fluid.
10	62	F	Diverticulum	Right flank pain radiating to RIF, bloating for 2 weeks.	US: mural thickening near ileocecal valve. Hydrosonography: segmental appendiceal hypoechoic thickening (9.2 mm) with adjacent fat stranding. Pelvic CT: inflammatory cecal/ascending colon changes. Colonoscopy: bulging appendiceal orifice. Repeat CT: 10 mm appendix, 3 mm wall thickening, adjacent 8–9 mm nodule.

Abbreviations: CT, computed tomography; LAMN, low-grade appendiceal mucinous neoplasm; MRI, magnetic resonance imaging; RIF, right iliac fossa; RLQ, right lower quadrant; US, ultrasonography.

**Table 2:** Intraoperative findings, surgical management, follow-up, and key pathology.

Case	Final Diagnosis	Intra-Op Findings	Procedure	Follow-Up	Key Pathology
1	Mucinous cystadenoma	Markedly enlarged, tense appendix.	Laparoscopic appendectomy	Uneventful	Mucin, fibrino-leukocytic exudate, flattened mucosa with focal pseudostratification and mild dysplasia; no invasion or malignancy.
2	Mucinous cystadenoma	Dilated tumor-like appendix; base suitable for appendectomy.	Laparoscopic appendectomy	Uneventful	Mucinous cystadenoma with focal papillary pattern and mild dysplasia; acellular mural/serosal mucin; CK7+/CK20+; Ki-67 10–20%.
3	LAMN, pTis	The appendix was dilated up to 2 cm from the middle third to its cecal insertion; the appendiceal tip had a normal appearance, was subserosal, and was located almost subhepatically.	Laparoscopic appendectomy + cecal wall resection	Uneventful	Mucin-filled dilated lumen, thinned wall, submucosal fibro-hyalinosis, minimal atypia, and acellular submucosal mucin lakes; no epithelial cells in lakes.
4	LAMN	Incarcerated right obturator hernia with entrapped ileal loop; appendiceal tumor found incidentally.	Open appendectomy	Uneventful	Thin wall, denuded surface epithelium, markedly dilated lumen with abundant acellular mucin; focal mural mucin infiltration.
5	LAMN, pTis	Dilated appendix with broad base and cecal intussusception-like appearance.	Laparoscopic right hemicolectomy	Uneventful	Low-grade atypia with endoluminal micropapillae; Ki-67 about 10% (15% in hotspots); intramural acellular mucin extending into probable diverticulum; no invasion.
6	LAMN	3.5 cm cystic lesion in the proximal appendix, invaginated into the cecum.	Open ileocecal resection	Uneventful	Histology consistent with LAMN.
7	LAMN	Dilated appendix with minimal local inflammatory changes.	Laparoscopic appendectomy	Uneventful	Mucinous epithelium with low-grade atypia, mural mucin pools, and fibrosis; consistent with LAMN.
8	Adenocarcinoma, pT4bN0L0V1R1	Ceco-appendiceal tumor invading iliopsoas and adherent to testicular vessels; 3 cm node and ~50 mL purulent collection with tumor debris; femoral nerve partially resected.	Open right hemicolectomy + iliopsoas resection + hilar node biopsy	Oncology referral; 8 XELOX cycles; 1-year local recurrence resected; ongoing follow-up.	Mucinous adenocarcinoma (>50% extracellular mucin) infiltrating the colonic wall and reaching the resection margin (R1); proximal/distal margins negative; focal vascular emboli; no perineural invasion.
9	Stump appendicitis	Appendiceal stump about 2 cm, fibrin-coated and phlegmonous.	Laparoscopic stump appendectomy	Uneventful	Marked mucosal ulceration, intramural abscesses, edema, hemorrhage, congestion, and dense PMN infiltrate; fibrino-leukocytic serosal exudate.
10	Diverticulum	Dilated appendix with associated cystic change.	Laparoscopic appendectomy	Uneventful	Mucosal erosions, ulcerated mesoappendiceal diverticula, suppuration extending into adjacent fat, and fibrino-leukocytic serosal deposits.

Abbreviations: LAMN, low-grade appendiceal mucinous neoplasm; PMN, polymorphonuclear; XELOX, capecitabine plus oxaliplatin.

**Table 3:** Distinguishing imaging characteristics of acute appendicitis and rare appendicular pathologies.

Pathology	Typical Imaging Findings	Distinguishing Features Compared with Typical Acute Appendicitis
Acute appendicitis	Enlarged appendix with wall thickening, periappendiceal fat stranding, mural hyperenhancement, and possible appendicolith or inflammatory free fluid.	Reference inflammatory pattern; findings are usually dominated by acute inflammation and match an acute clinical syndrome.
Mucinous cystadenoma	Cystic or tubular appendiceal dilatation with fluid content, thin wall, possible mural calcifications, and limited surrounding inflammatory change.	More mucocele-like than inflammatory, often with marked appendiceal enlargement and relatively little periappendiceal fat stranding.
Low-grade appendiceal mucinous neoplasm (LAMN)	Marked appendiceal dilatation with mucinous content, broad appendiceal base, possible cecal involvement or intussusception-like appearance, thin wall, and usually limited inflammatory changes.	Raised suspicion with marked dilatation, mucocele-type morphology, base involvement, or a target-like/invagination pattern rather than a classic inflamed appendix.
Appendiceal adenocarcinoma	Mass-forming or infiltrative appendiceal/ceco-appendiceal lesion, irregular wall abnormality, surrounding abscess or collection, regional lymphadenopathy, and possible invasion of adjacent structures.	Suggests a more aggressive process than simple appendicitis, especially when there is a tumoral mass, nodal disease, persistent collection, or extension into neighboring tissues.
Appendicular diverticulum	Focal appendiceal wall abnormality or diverticular outpouching with adjacent inflammatory change and sometimes subtle periappendiceal nodular or cystic findings.	May mimic appendicitis, but the abnormality can be more focal or atypical rather than diffuse appendiceal inflammation.
Stump appendicitis	Residual tubular appendiceal stump with mural thickening, surrounding fat stranding, and possible free fluid or collection in a patient with previous appendectomy.	The key distinguishing feature is an inflamed appendiceal remnant in the setting of prior appendectomy.

## Data Availability

No new data were created or analyzed in this study. Data sharing is not applicable to this article.
